# Deciphering the role of Saliva in COVID 19: A global cross-sectional study on the knowledge, awareness and perception among dentists

**DOI:** 10.1186/s12903-023-03152-2

**Published:** 2023-06-26

**Authors:** Selvakumar Kritika, Sekar Mahalaxmi, N Srinivasan, Jogikalmat Krithikadatta

**Affiliations:** 1 Department of Conservative Dentistry and Endodontics, SRM Dental College, Ramapuram, SRM Institute of Science & Technology, Ramapuram Campus, Bharathi Salai, Ramapuram, Chennai, Tamil Nadu 600089 India; 2 Specialist Endodontist, Hamad Dental Center, Hamad Medical Corporation, Doha, Qatar; 3 Department of Cariology and Department of Conservative Dentistry and Endodontics, Saveetha Dental College and Hospitals, Chennai, Tamil Nadu 600077 India

**Keywords:** Saliva, Coronavirus, Dentists, Diagnosis, Knowledge, Awareness, Perception

## Abstract

**Objectives:**

The global pandemic outbreak of the coronavirus has instilled the quest amongst researchers on the expedited need for the early detection of viral load. Saliva is a complex oral biological fluid which not only causes the disease transmission but can be an effective alternative sample for detection of SARS-CoV2. This provides an ideal opportunity for dentists to be the frontline healthcare professionals who can collect the salivary samples; however the awareness of this amongst dentists is uncertain. Hence the aim of this survey was to evaluate the knowledge, perception and awareness of the role of saliva in detecting the SARS-CoV2 among dentists worldwide.

**Methods:**

The online questionnaire comprising of 19 questions was shared to 1100 dentists worldwide and a total of 720 responses was collected. The data was tabulated, statistically analysed using the non- parametric Kruskal-Wallis test (p < 0.05). Based on the principal component analysis, 4 components (knowledge about virus transmission, perception about SARS-CoV2 virus, awareness on the sample collection and knowledge about prevention of the virus) were obtained which was compared with the 3 independent variables (years of clinical experience, occupation and region).

**Results:**

A statistically significant difference was observed in the awareness quotient amongst the dentists with 0–5 years and greater than 20 years of clinical experience. In terms of the occupation, a significant difference was noted when comparing the postgraduate students to practitioners knowledge about the virus transmission. A highly significant difference was seen on comparing academicians and postgraduate students and also between academicians and practitioners. No significant difference was evidenced amongst the different regions, however the mean score was in the range of 3-3.44.

**Conclusion:**

This survey highlights the deficiency in the knowledge, perception and awareness among dentists worldwide.

**Supplementary Information:**

The online version contains supplementary material available at 10.1186/s12903-023-03152-2.

## Clinical Significance

The imperative need for updating the knowledge, awareness and perception among dentists worldwide on the role of saliva in COVID 19 as it not only transmits the disease but is also a promising diagnostic aid.

**Supplementary Information:**

The online version contains supplementary material available at 10.1186/s12903-023-03152-2.

## Introduction

“Oral cavity is the mirror of the body” with saliva being a double edged weapon, which is not only a causative but also a curative factor for various diseases. COVID 19 is an infectious rapidly spreading pandemic disease caused by the SARS-CoV2 virus affecting the people worldwide. The SARS-CoV2 are positive single stranded enveloped virus of the Nidovirales order belonging to the Coronaviridae family [1]. Literature reports four genera of virus namely alpha, beta, gamma, delta wherein the SARS-CoV2 belongs to the lineage B of β- coronavirus genera with a genome size of 29.9 kb [2–4]. The diversification and evolutionary changes in the SARS-CoV2 genome in the geographic dissemination process led to increased genetic variation and distinct mutations [5, 6]. Symptoms of this dreadful disease ranges from mild cough to serious life threatening respiratory illness. According to the latest reports from the World Health Organisation (WHO), about 53,08,96,347 people worldwide have been affected by COVID 19. WHO has reported that SARS-CoV2 virus primarily spreads through salivary droplets or respiratory secretions from an infected individual. Transmission of the viral droplets can occur via coughing, sneezing or talking in close contact.

In the field of dentistry, the aerosol generated during dental procedures may contain these salivary droplets from covid patients/carriers undergoing dental treatment contributing to the transmission of this virus. About 1,716 to 17,306 cases with 605 mortalities have been reported due to the cross- contamination of the 2019 novel corona virus to healthcare providers across the world [7]. On the other hand, the presence of the SARS-CoV2 virus in saliva facilitates their early detection. Currently used method for diagnosis of the virus includes nasopharyngeal swab and oral swabs, however it causes discomfort to the individuals during the sample collection. Since the dentists deal with the oral cavity, they will be the ideal professionals for collecting the samples, however their knowledge in this aspect is inadequate. Hence this survey was undertaken to assess the knowledge, perception and awareness of the role of saliva in detecting the virus among dentists.

## Materials and methods

### Formulation of questionnaire

An online survey questionnaire was designed comprising of 28 questions which was evaluated for content validity and face validity and a pilot study was conducted. A panel of field experts reviewed the 28 questionnaire items, assessing their relevance, clarity, and comprehensiveness. The experts’ valuable feedback was incorporated into the questionnaire, to refine and validate the contents. Subsequently, a pilot study was conducted, where participant feedback on item relevance and clarity was carefully analysed. Based on the pilot study findings, a validated questionnaire was formulated, consisting of 19 questions that improved face validity. The validated questionnaire consisting of 19 questions based on the pilot study was formulated. The study protocol was presented to the Institutional Review Board and approval was obtained (SRMU/M&HS/SRMDC/2020/S/018).

### Study participants

The survey questionnaire was created using the free- access google forms application and the link (https://forms.gle/WqzaKy5uecHyRpeX6) was circulated amidst 1100 dentists worldwide. An informed consent was obtained from the dentists for their voluntary participation in the survey.

### Data collection

The data was collected from June 2020 to December 2020, where both convenience sampling and snowball sampling was used for maximum participation of dentists globally. The questionnaire was divided into 2 sections, where section 1 comprised of demographic details and section 2 was structured with the 19 questions assessing the knowledge, perception, awareness and prevention of SARS-CoV2 virus. A total of 720 responses was obtained from dentists worldwide and the data was tabulated. With a response rate of approximately 65%, the obtained sample size of 720 respondents can be considered substantial for drawing meaningful conclusions. This sample size provides a reasonable representation of the target population and allows for statistically significant analyses, considering the general guideline that larger sample sizes tend to yield more reliable results.

### Statistical analysis

The data were statistically analysed using the SPSS software version 25. Descriptive statistical anlaysis was done. Principal component analysis was used to reduce the dimensions of the 12 questions, which were based on the likert scale into 4 components namely knowledge about virus transmission in component 1 (C1), perception about SARS-CoV2 virus in component 2 (C2), awareness on the sample collection in component 3 (C3) and knowledge about prevention/inhibition of the virus in component 4 (C4). Based on Eigen value, the four components were retained (KMO measure = 0.66, Barthlett test of sphericity p < 0.001). The cumulative variance was 50.32%. Kruskal Wallis test was employed for intergroup analysis to compare the 4 dependent variables with the 3 independent variables namely years of clinical experience, occupation and region.

## Results

The results of the comparative evaluation between the independent and dependent variables is as follows:

### Years of clinical experience

The mean and standard deviation values of the 4 components by the respondents with varying years of clinical experience is tabulated in Table 1. Among the four components, there was a statistically significant difference only with C3 (p = 0.012). On further pairwise comparison within the 4 categories of years of clinical experience, significant difference between category 1 and 4 was found (p = 0.042).

**Table 1:** Intergroup comparison of years of clinical experience based on the four components

YEARS OF CLINICAL EXPERIENCE	C1	C2	C3	C4
0–5 YEARS	3.67 ± 0.531^a^	3.29 ± 0.661^a^	2.70 ± 0.843^a^	3.22 ± 0.513^a^
6–10 YEARS	3.58 ± 0.561^a^	3.20 ± 0.697^a^	2.70 ± 0.913^a,b^	3.30 ± 0.543^a^
11–20 YEARS	3.53 ± 0.647^a^	3.14 ± 0.691^a^	2.93 ± 0.908^a,b^	3.32 ± 0.567^a^
ABOVE 20 YEARS	3.52 ± 0.744^a^	3.24 ± 0.771^a^	3.06 ± 0.900^b^	3.29 ± 0.564^a^

^a,b^ Different alphabetical superscript indicates the statistical significant difference in the mean(SD) scores

### Occupation

Components 1 and 3 (p = 0.003) were found to be statistically significant with C1 being highly significant (p = 0.000). Among the subcategories, the level of significance can be represented as (Table 2) :

C1 - postgraduate students ≥ academicians ≥ practitioners, with significant difference between postgraduate student and practitioners.

C3 – academicians > postgraduate students ≥ practitioners.

**Table 2:** Intergroup comparison of occupation based on the four components

OCCUPATION	C1	C2	C3	C4
ACADEMICIAN	3.62 ± 0.613^a,b^	3.27 ± 0.750^a^	2.95 ± 0.920^a^	3.33 ± 0.592^a^
POSTGRADUATE STUDENT	3.75 ± 0.523^a^	3.26 ± 0.636^a^	2.72 ± 0.830^a,b^	3.30 ± 0.496^a^
PRACTITIONER	3.53 ± 0.574^b^	3.22 ± 0.671^a^	2.68 ± 0.870^b^	3.19 ± 0.509^a^

^a,b^ Different alphabetical superscript indicates the statistical significant difference in the mean(SD) scores

### Region

There was no statistical difference among the different regions (Africa, Asia, Central America, Eastern Europe, European Union, Middle East, North America, Oceania, South America, Caribbean, Other Asian Countries) when evaluating across the various components with the mean score in the range of 3-3.44.

## Discussion

Saliva is a multifaceted homeostatic concoction of the oral biological fluid which contains salivary gland secretion, sputum and/or mucosal transudate and gingival crevicular fluid. About 600ml of saliva is generated by a normal adult in a day. The hidden capability of saliva has been unleashed in the early detection of any bacterial, viral or systemic diseases wherein it proves to play a pivotal role in the isolation of proteins, peptides and viral assessment via molecular assays. In the recent past, salivary biomarkers have been found to be a valuable tool for the detection of diseases such as diabetes, breast cancer, lung cancer, oral cancer, dental caries and periodontal diseases [8, 9]. Saliva- based antibody assessments at the proteomic levels have been carried out for the detection of viruses namely hepatitis (A, B, C), HIV-1, measles, rubella, mumps and vesicular stomatitis [10]. In this context, the results of our survey indicate that 27.1% of dentists are aware that salivary cystatins and salivary proteins may act as a protective barrier inhibiting the viral replication of SARS-CoV2 [11, 12].

Literature reports saliva as an alternate sample for the detection of SARS-CoV2. In the systematic review by Cañete et al.[13], the high viral load present in saliva in symptomatic patients can be detected at a higher sensitivity rate and is comparable to the conventional nasopharyngeal swab (NPS) sample collection method. On the other hand, various studies prove the viability and detectability of SARS- CoV2 virus in the salivary samples even when the viral load is low [14–16]. Zhu et al. [17] reported that saliva had 86.4% sensitivity and 97% specificity when assessed in 944 patients from 12 independent cohorts. The sensitivity and specificity are relatively comparable to the NPS in symptomatic cases with high viral load and can be considered moderately acceptable in asymptomatic cases with low viral load [18, 19]. Further, a 97.4% concordance index between the respiratory tract and salivary samples were observed with a kappa of 0.87% [20].

The results of this survey succinctly revealed that less than 50% of the dentists worldwide selected the correct response when assessed in terms of knowledge about virus transmission (C1), perception about SARS-CoV2 virus (C2), awareness about the sample collection (C3) and knowledge about prevention of the virus (C4). A statistically significant difference was noted in the awareness quotient among the dentists with less than 5 years and greater than 20 years of clinical experience. The dentists with greater than 20 years of clinical experience were aware about the sample collection method when compared with the other categories. In terms of the occupation, a highly significant difference was observed between the postgraduate students and practitioners on the knowledge about the virus transmission. Further, the awareness of academicians showed a statistically significant difference when compared to the postgraduate students and practitioners.

According to the analysis by the O*Net Bureau of Labour Statistics of the USA and New York Times, dentists are highly prone to acquire SARS-CoV2 virus [21]. The nature of their work entails increased production of aerosols and the close proximity to salivary bioaerosols augments the transmission of the virus. Our survey included dentists worldwide who treat a minimum of 5 patients a day and 90.8% of the dentists participating in our survey knew that dentists may acquire the virus from salivary bioaerosols. Further, dentists may also acquire the pathogen during the process of taking an intraoral periapical radiograph (IOPA) as it may induce a gag reflex or stimulate a cough, which was acknowledged by 79.5% of our surveyees. Therefore in the current clinical scenario, due to the increased prevalence of the transmissible SARS-CoV2 virus, an orthopantamogram is recommended [22].

Studies reveal that approximately three thousand salivary droplet nuclei are produced by each cough or during a conversation in close contact at a distance of 1–3 m for 5 min [23, 24]. Stadnytskyi et al. [25] demonstrated that the suspension of airborne droplets generated during normal speech can remain viable for tens of minutes or longer enabling the transmission of the virus. However, 52.9% respondents were unaware of the jeopardy caused due to 1–3 m of close contact, as the aerosols can be transmitted to long distances (> 1 m) which is dictated by the airborne small infectious salivary droplets [26]. Though the incubation period for the viral infection is 14 days, Tajima et al. [27] demonstrated that the SARS-CoV2 viral strains survived in saliva for 37 days post infection in asymptomatic cases, which only 27% of the dentists worldwide were aware of.

Literature reports reveal that the expression of the virus affecting the ACE2 + receptors is higher in the tongue followed by the salivary glands when compared to the lungs and other organs of the body, which was discerned by only one fourth of our survey population [28]. Further only 18.9% respondents were aware that ACE2 + receptors of the salivary glands are the portal of entry for the attachment of the SARS-CoV2 virus, subsequently leading to their replication triggering acute or chronic sialadenitis leading to xerostomia/ hyposalivation [29]. Zhu et al. [17] demonstrated that during the onset of symptoms (first week), a peak viral load of 10^4^- 10^6^ viral copies per mL could be detected in saliva. Liu et al. [30] proved in rhesus macaques, that the major viral source of SARS coronavirus is the ACE2 + receptors of the salivary glands where it could be detected within 48 h post infection. Wang et al. [31] detected the presence of SARS coronavirus RNA in human saliva at 4.8 days indicating a high early detection rate and suggested that saliva is an ideal specimen for early diagnosis of SARS-CoV2 virus. Wyllie et al. [14] reported that saliva is a reliable sample which is more sensitive and consistent than the NPS for detection of the virus alleviating the testing demands of COVID 19 (as opined by 51.7% of our respondents). To et al. [32] proved that a consistent detection of the SARS-CoV2 virus in the salivary samples was elicited in 11 patients admitted since the first day of hospitalisation. On the contrary, in our survey only 22.4% respondents confirmed that saliva is more sensitive and consistent than NPS.

The inherent advantages of the salivary sample collection, such as, being a non-invasive method, can be self-collected, cheap and does not require a healthcare worker for collection of the sample facilitate its use in the current pandemic scenario [33]. Dentists can play a pivotal role as they could be mediators for effective salivary sample collection, promoting the early detection of SARS-CoV2 virus which was acknowledged by 64.4% of our respondents [34]. On the other hand, 48.1% of our respondents were aware that salivary samples could be self-collected and 68.5% respondents were of the view that a trained healthcare worker is not essential for the salivary sample collection, where the shortage of personal protective equipment and risk of transmission of the virus can be surmounted [35]. To et al. [32] reported a 2.26–2.59 fold decrease in the time and cost required for salivary sample collection when compared to NPS. Salivary sample collection methods include spitting into a collection tube, coughing out saliva, drooling of secretions from parotid glands, most of which was unknown to 99% of the dentists who participated in our survey [36].

In the clinical scenario, Seneviratne et al. [37] claimed that the use of preprocedural mouth rinse reduced the transmission of COVID 19. However, only 29.9% of our respondents knew that a pre-procedural mouth rinse advocation should be avoided prior to salivary sample collection. However, it is to be noted that the limitations include that the perspective of respondents might have changed with an increase in percentage in the current clinical practice. This discrepancy could be due to the fact that the survey was conducted during the first wave of COVID 19. Though the sample size is large enough for a survey, a more equal distribution of respondents from various geographic regions may provide a more meaningful interpretation of the results.

On May 9th 2020, the first salivary collection device and diagnostic kit for detection of SARS-CoV2 virus in salivary samples was approved by FDA, which was not known to many of the respondents (75.9%) [38]. Nowadays, numerous approved kits have been employed for salivary sample collection. Pinilla et al. [39] reported that SARS-CoV2 antibodies are prevalent upto 15 months in saliva. The spike in IgG and IgA in saliva serves as the ‘first line of defense’ against the SARS-CoV2 virus. In the future, researchers can focus on driving these salivary antibodies towards yielding a vaccine to combat the dreadful SARS-CoV2 virus, which was suggested by 54.8% of our respondent dentists.

Despite the numerous benefits of saliva, the knowledge, perception and awareness amongst dentists worldwide on the role of saliva as a diagnostic tool is limited. Literature evidences reveal that saliva could be an effective alternative to nasopharyngeal swabs, however its routine use in clinical practice has not been widely accepted due to the limited number of clinical trials employing the use of salivary samples in the detection of SARS-CoV2 [13, 14, 40]. Interestingly, it is evident from our survey results that the dentists worldwide have accepted the use of saliva as a diagnostic tool (average score 7–8). Further studies and clinical trials need to be undertaken to establish saliva as the first line diagnostic tool against SARS-CoV2 virus.

## Conclusion

In the present survey the lack of knowledge, perception and awareness amongst dentists on the role of saliva has been brought to light. Hence, the stakeholders and government organizations must provide adequate training and adopt measures to emphasize the role of saliva as a promising diagnostic aid in the early detection and management of the SARS-CoV2 virus.

## Electronic supplementary material

Below is the link to the electronic supplementary material.

Supplementary Material 1

## Data Availability

All data generated or analysed during this study are included in this published article (and its Supplementary Information files).

## References

[1] Su S, Wong G, Shi W, Liu J, Lai AC, Zhou J, Liu W, Bi Y, Gao GF (2016). Epidemiology, genetic recombination, and pathogenesis of coronaviruses. Trends Microbiol.

[2] Perlman S, Netland J (2009). Coronaviruses post-SARS: update on replication and pathogenesis. Nat Rev Microbiol.

[3] Letko M, Marzi A, Munster V (2020). Functional assessment of cell entry and receptor usage for SARS-CoV-2 and other lineage B betacoronaviruses. Nat Microbiol.

[4] Wu F, Zhao S, Yu B, Chen YM, Wang W, Song ZG, Hu Y, Tao ZW, Tian JH, Pei YY, Yuan ML (2020). A new coronavirus associated with human respiratory disease in China. Nature.

[5] Korber B, Fischer WM, Gnanakaran S, Yoon H, Theiler J, Abfalterer W, Hengartner N, Giorgi EE, Bhattacharya T, Foley B, Hastie KM. Tracking changes in SARS-CoV-2 spike: evidence that D614G increases infectivity of the COVID-19 virus, Cell 182 (2020) 812–27. 10.1016/j.cell.2020.06.043 PMC7332439 32697968

[6] Kumar BK, Venkatraja B, Prithvisagar KS, Rai P, Rohit A, Hegde MN, Karunasagar I, Karunasagar I. Mutational analysis unveils the temporal and spatial distribution of G614 genotype of SARS-CoV-2 in different Indian states and its association with case fatality rate of COVID-19. (2020). *bioRxiv*.

[7] Sant’Ana G, Imoto AM, Amorim FF, Taminato M, Peccin MS, Santana LA, Göttems LBD, Camargo EB (2020). Infection and death in healthcare workers due to COVID-19: a systematic review. Acta Paul Enferm.

[8] Khurshid Z, Zafar MS, Khan RS, Najeeb S, Slowey PD, Rehman IU (2018). Role of salivary biomarkers in oral cancer detection. Adv Clin Chem.

[9] Khurshid Z, Zafar M, Khan E, Mali M, Latif M (2019). Human saliva can be a diagnostic tool for Zika virus detection. J Infect Public Health.

[10] Zhang CZ, Cheng XQ, Li JY, Zhang P, Yi P, Xu X, Zhou XD (2016). Saliva in the diagnosis of diseases. Int J Oral Sci.

[11] Collins AR, Grubb A, Cystatin D (1998). A natural salivary cysteine protease inhibitor, inhibits coronavirus replication at its physiologic concentration. Oral Microbiol Immunol.

[12] Farshidfar N, Hamedani S (2020). Hyposalivation as a potential risk for SARS-CoV‐2 infection: inhibitory role of saliva. Oral Dis.

[13] Cañete MG, Valenzuela IM, Garcés PC, Massó IC, González MJ, Providell SG (2021). Saliva sample for the massive screening of SARS-CoV-2 infection: a systematic review. Oral Surg Oral Med Oral Pathol Oral Radiol.

[14] Wyllie AL, Fournier J, Casanovas-Massana A, Campbell M, Tokuyama M, Vijayakumar P, Geng B, Muenker MC, Moore AJ, Vogels CB, Petrone ME (2020). Saliva is more sensitive for SARS-CoV-2 detection in COVID-19 patients than nasopharyngeal swabs. N Engl J Med.

[15] Tutuncu EE, Ozgur D, Karamese M (2021). Saliva samples for detection of SARS-CoV‐2 in mildly symptomatic and asymptomatic patients. J Med Virol.

[16] Teo AKJ, Choudhury Y, Tan IB, Cher CY, Chew SH, Wan ZY, Cheng LTE, Oon LLE, Tan MH, Chan KS, Hsu LY (2021). Saliva is more sensitive than nasopharyngeal or nasal swabs for diagnosis of asymptomatic and mild COVID-19 infection. Sci Rep.

[17] Zhu J, Guo J, Xu Y, Chen X (2020). Viral dynamics of SARS-CoV-2 in saliva from infected patients. J Infect.

[18] Pasomsub E, Watcharananan SP, Boonyawat K, Janchompoo P, Wongtabtim G, Suksuwan W, Sungkanuparph S, Phuphuakrat A (2021). Saliva sample as a non-invasive specimen for the diagnosis of coronavirus disease 2019: a cross-sectional study. Clin Microbiol Infect.

[19] Chen JHK, Yip CCY, Poon RWS, Chan KH, Cheng VCC, Hung IFN, Chan JFW, Yuen KY, To KKW (2020). Evaluating the use of posterior oropharyngeal saliva in a point-of-care assay for the detection of SARS-CoV-2, Emerg. Microbes Infect.

[20] Iwasaki S, Fujisawa S, Nakakubo S, Kamada K, Yamashita Y, Fukumoto T, Sato K, Oguri S, Taki K, Senjo H (2020). Sugita. Comparison of SARS-CoV-2 detection in nasopharyngeal swab and saliva. J Infect.

[21] Gamio L. The workers who face the greatest coronavirus risk. New York Times Mar 15(2020). https://www.nytimes.com/interactive/2020/03/15/business/economy/coronavirus-worker-risk.html.

[22] Meng L, Hua F, Bian Z (2020). Coronavirus disease 2019 (COVID-19): emerging and future challenges for dental and oral medicine. J Dent Res.

[23] Kohn WG, Collins AS, Cleveland JL, Harte JA, Eklund KJ, Malvitz DM. Guidelines for infection control in dental health-care settings-2003. 52 (2003) 17. 14685139

[24] Fineberg HV. Rapid expert consultation on the possibility of bioaerosol spread of SARS-CoV-2 for the COVID-19 pandemic, the National Academies of Sciences, Engineering, and Medicine. Apr 1 (2020).

[25] Stadnytskyi V, Bax CE, Bax A, Anfinrud P (2020). The airborne lifetime of small speech droplets and their potential importance in SARS-CoV-2 transmission. Proc Natl Acad Sci.

[26] Fennelly KP, Martyny JW, Fulton KE, Orme IM, Cave DM, Heifets LB (2004). Cough-generated aerosols of Mycobacterium tuberculosis: a new method to study infectiousness. Am J Respir Critic Care Med.

[27] Tajima Y, Suda Y, Yano K (2020). A case report of SARS-CoV-2 confirmed in saliva specimens up to 37 days after onset: proposal of saliva specimens for COVID-19 diagnosis and virus monitoring. J Infect Chemother.

[28] Xu H, Zhong L, Deng J, Peng J, Dan H, Zeng X, Li T, Chen Q (2020). High expression of ACE2 receptor of 2019-nCoV on the epithelial cells of oral mucosa. Int J Oral Sci.

[29] Wang C, Wu H, Ding X, Ji H, Jiao P, Song H, Li S, Du H (2020). Does infection of 2019 novel coronavirus cause acute and/or chronic sialadenitis?. Med Hypotheses.

[30] Liu L, Wei Q, Alvarez X, Wang H, Du Y, Zhu H, Jiang H, Zhou J, Lam P, Zhang L, Lackner A (2011). Epithelial cells lining salivary gland ducts are early target cells of severe acute respiratory syndrome coronavirus infection in the upper respiratory tracts of rhesus macaques. J Virol.

[31] Wang WK, Chen SY, Liu IJ, Chen YC, Chen HL, Yang CF, Chen PJ, Yeh SH, Kao CL, Huang LM, Hsueh PR (2004). Detection of SARS-associated coronavirus in throat wash and saliva in early diagnosis. Emerg Infect Dis.

[32] To KKW, Tsang OTY, Yip CCY, Chan KH, Wu TC, Chan JMC, Leung WS, Chik TSH, Choi CYC, Kandamby DH, Lung DC (2020). Consistent detection of 2019 novel coronavirus in saliva. Clin Infecti Dis.

[33] Vinayachandran D, Balasubramanian S (2020). Salivary diagnostics in COVID-19: future research implications. J Dent Sci.

[34] Fallahi HR, Keyhan SO, Zandian D, Kim SG, Cheshmi B (2020). Being a front-line dentist during the Covid-19 pandemic: a literature review. Maxillofac Plast Reconstr Surg.

[35] Santosh TS, Parmar R, Anand H, Srikanth K, Saritha M (2020). A review of salivary diagnostics and its potential implication in detection of Covid-19. Cureus.

[36] Fini MB (2020). Oral saliva and COVID-19. Oral Oncol.

[37] Seneviratne CJ, Balan P, Ko KKK, Udawatte NS, Lai D, Ng DHL, Venkatachalam I, Lim KS, Ling ML, Oon L, Goh BT (2021). Efficacy of commercial mouth-rinses on SARS-CoV-2 viral load in saliva: randomized control trial in Singapore. Infection.

[38] US Food and Drug Administration. Coronavirus (COVID-19) update: FDA authorizes first diagnostic test using at-home collection of saliva specimens. May 8 (2020).

[39] Pinilla YT, Heinzel C, Caminada LF, Consolaro D, Esen M, Kremsner PG, Held J, Kreidenweiss A, Fendel R (2021). SARS-CoV-2 antibodies are persisting in saliva for more than 15 months after infection and become strongly boosted after vaccination. Front Immunol.

[40] Li Q, Guan X, Wu P, Wang X, Zhou L, Tong Y, Ren R, Leung KS, Lau EH, Wong JY, Xing X (2020). Early transmission dynamics in Wuhan, China, of novel coronavirus–infected pneumonia. N Engl J Med.

