# Supplementary material for: Deciphering the role of Saliva in COVID 19: A global cross-sectional study on the knowledge, awareness and perception among dentists
Source: BMC Oral Health. 2023 Jun 26;23:424. doi: 10.1186/s12903-023-03152-2 (PMC10294381; doi:10.1186/s12903-023-03152-2)
Supplement: Supplementary file 1 — Supplementary Material 1 [file 12903_2023_3152_MOESM1_ESM.pdf]

SURVEY QUESTIONNAIRE

Deciphering the Role of Saliva in COVID 19: A Global Survey on the Knowledge, Awareness and Perception among Dentists.

Age (in completed years):
Gender:

- Male
- Female

Area of Specialization:

- General Practitioner
- Endodontics
- Restorative Dentistry
- Prosthodontics
- Orthodontics
- Periodontics
- Pedodontics
- Oral and Maxillofacial Surgery
- Community Dentistry
- Oral Medicine and Radiology
- Oral Pathology
- PhD

Years of Clinical Experience:

- 0-5 years
- 6-10 years
- 11-20 years
- Above 20 years

Occupation : (multiple options)

- Postgraduate student
- Academician
- Practitioner
- Researcher

Region:

- Africa
- India
- Other Asian Countries
- Central America
- Eastern Europe
- European Union
- Middle East
- North America
- Oceania
- South America
- The Caribbean

Email:

1. What is the average number of patients seen per day in your dental practice?
    a) Less than 5 patients
    b) 5-10 patients
    c) 11-20 patients
    d) 21-40 patients
    e) >40 patients

2. Will the SARS CoV-2 virus be present in the saliva of a COVID -19 infected patient?
    a) Yes
    b) No
    c) I don’t know

3. Do you believe that dentists may acquire the SARS CoV-2 virus infection from salivary bioaerosols?
    a) Strongly disagree
    b) Disagree
    c) Neither agree nor disagree
    d) Agree
    e) Strongly agree

4. Do you think dentists can acquire the SARS CoV-2 virus when conversing with a patient in a radius of 2metres?
    a) Strongly disagree
    b) Disagree
    c) Neither agree nor disagree
    d) Agree
    e) Strongly agree

5. Do you think a salivary sample is as effective as nasopharyngeal swab in early detection of SARS CoV-2 virus?
    a) Strongly disagree
    b) Disagree
    c) Neither agree nor disagree
    d) Agree
    e) Strongly agree

6. Preprocedural mouth rinse is required prior to salivary sample collection.
    a) Strongly disagree
    b) Disagree
    c) Neither agree nor disagree
    d) Agree
    e) Strongly agree

7. Dental healthcare workers are at risk of acquiring SARS CoV-2 virus during the process of taking an Intraoral radiograph.
    a) Strongly disagree
    b) Disagree
    c) Neither agree nor disagree
    d) Agree
    e) Strongly agree

8. The highest expression of ACE2+ receptors in the oral cavity is in the
    a) Tongue
    b) Salivary glands
    c) Gingiva

d) Buccal mucosa

9. SARS CoV-2 virus could survive in the saliva for up to 1 month.
    a) Strongly disagree
    b) Disagree
    c) Neither agree nor disagree
    d) Agree
    e) Strongly agree

10. Saliva may provide a protective mechanism against SARS CoV-2 virus.
    a) Strongly disagree
    b) Disagree
    c) Neither agree nor disagree
    d) Agree
    e) Strongly agree

11. Is hyposalivation one of the symptoms of COVID 19 disease?
    a) Yes
    b) No
    c) I don’t know

12. Do you think salivary sample is more sensitive and consistent than nasopharyngeal swabs ?
    a) Strongly disagree
    b) Disagree
    c) Neither agree nor disagree
    d) Agree
    e) Strongly agree

13. Is there any approved diagnostic test that uses salivary sample for detecting SARS CoV-2 (COVID- 19) virus?
    a) Yes
    b) No
    c) I don’t know

    Answer: Yes

14. Do you think dental healthcare workers can collect the salivary samples for SARS CoV-2 virus screening?

a) Strongly disagree
b) Disagree
c) Neither agree nor disagree
d) Agree
e) Strongly agree

15. Salivary samples can be self- collected by the patient himself.
   a) Strongly disagree
   b) Disagree
   c) Neither agree nor disagree
   d) Agree
   e) Strongly agree

16. Which of the following is/are the approach(es) for salivary sample collection.
   a) Spitting into a collection tube
   b) Coughing out saliva
   c) Drooling
   d) Parotid gland secretions

17. A trained healthcare worker is required for salivary sample collection.
   a) Strongly disagree
   b) Disagree
   c) Neither agree nor disagree
   d) Agree
   e) Strongly agree

18. Can salivary antibodies play a role in yielding a vaccine to combat COVID 19 in the future?
   a) Strongly disagree
   b) Disagree
   c) Neither agree nor disagree
   d) Agree
   e) Strongly agree

19. In the context of COVID 19, rate the role of saliva :

*Score 0 indicates bane and score 10 indicates boon

Score:
